# 

**Published:** 1899-12

**Authors:** 


*Fifty-Fifth Year.*

VOL. LV.
WHOLE NUMBER,
DCXXXVII

DECEMBER, 1899.

New Series.
VOL. XXXIX.
No. 5.

### BUFFALO MEDICAL JOURNAL

LIBRARY
Indexed

Established 1845 by AUSTIN FLINT, M. D.

EDITOR:

WILLIAM WARREN POTTER, M. D.

ASSISTANT EDITORS:

WILLIAM C. KRAUSS, M. D.
NELSON W. WILSON, M. D.

ASSOCIATE EDITORS:

JAMES WRIGHT PUTNAM, M. D.
ERNEST WENDE, M. D.
JOHN PARMENTER, M. D.
ALVIN A. HUBBELL, M. D.
JOHN A. MILLER, PH. D.
MAUD J. FRYE, M. D.

LIBRARY
SURGEON GENERAL'S OFFI
DEC. 4--1899

EUROPEAN AGENCY, J. B. BAILLIERE & FILS, 19 Rue Hautefeuille, Paris.
*Subscription, if paid in advance, $2.00; otherwise, $2.50. Foreign subscription, $2.50.*
Copyright 1899, by WILLIAM WARREN POTTER, M. D.
Entered at the Post Office at Buffalo, N. Y., as second-class mail matter.

A. T. BROWN PRINTING HOUSE, BUFFALO, N. Y.

Table of Contents on Page V.

